# Pain, Opioids, and Functional Connectivity in Preterm Infants

**DOI:** 10.3390/children13020210

**Published:** 2026-01-31

**Authors:** Caterina Coviello, Lorenzo Frassineti, Camilla Fazi, Silvia Lori, Giovanna Bertini, Simona Montano, Simonetta Gabbanini, Clara Lunardi, Valentina Guarguagli, Antonio Lanata, Carlo Dani

**Affiliations:** 1 Division of Neonatology, Careggi University Hospital of Florence, 50134 Florence, Italy; covielloc@aou-careggi.toscana.it (C.C.); camilla.fazi@unifi.it (C.F.); giovanna.bertini@unifi.it (G.B.); montanos@aou-careggi.toscana.it (S.M.); clara.lunardi@unifi.it (C.L.); 2 Department of Information Engineering, Università degli Studi di Firenze, Via Santa Marta 3, 50139 Florence, Italy; lorenzo.frassineti@unifi.it (L.F.); valentina.guarguagli@unifi.it (V.G.); antonio.lanata@unifi.it (A.L.); 3 Neurophysiology Unit, Neuro-Musculo-Skeletal Department, Careggi University Hospital, 50134 Florence, Italy; silvia.lori@unifi.it (S.L.); gabbaninis@aou-careggi.toscana.it (S.G.); 4 Department of Neurosciences, Psychology, Drug Research and Child Health, Careggi University Hospital of Florence, 50134 Florence, Italy

**Keywords:** pain, functional connectivity, EEG, BAYLEY-III

## Abstract

**Aim:** To investigate the impact of pain on some electroencephalographic (EEG) features at term equivalent age (TEA) and, second, to assess if the proposed EEG analysis may be predictive of the neurodevelopmental outcome at 24 months corrected age. **Methodology:** Infants born < 32 weeks of gestational age, without major brain injury, were studied with an 8-channel EEG recording at TEA. The number of skin-breaking procedures from birth to the EEG recording was collected, as well as opioid administration. The following EEG-based indexes were investigated: Brain Simmetry Index (BSI) and Circular Omega Complexity (COC). Multivariate statistical analysis was performed. **Results:** Seventy-seven preterm newborns were enrolled. The multivariate models showed that higher pain exposure resulted in higher BSI, lower COC μ (mean), and lower COC values related to δ waves (all *p* < 0.05). Fentanyl was associated with increased BSI values related to α and β waves (all *p* < 0.05). Morphine showed a positive effect on BSI and a negative effect on OC μ and COC on all frequency bands (all *p* < 0.05). COC related to δ waves was positively associated with cognitive outcomes (*p* = 0.034). **Conclusions:** Pain and opioids might impact brain dynamics in preterm infants. Quantitative multivariate EEG indexes may be helpful to characterize the neurodevelopmental outcomes.

## 1. Introduction

Improved survival rates among preterm infants represent one of the major achievements of modern perinatal and neonatal medicine. Despite this progress, these children remain vulnerable to adverse neurodevelopmental outcomes [1]. In addition to major disabilities related to severe brain lesions, approximately 25–50% of infants born before 32 weeks of gestation develop neurodevelopmental disorders later in childhood, even in the absence of detectable abnormalities on conventional neuroimaging [2,3,4].

Between 24 and 40 weeks of gestation, the fetal brain undergoes a critical phase of growth and maturation. When preterm birth occurs, this rapid maturation overlaps with the exposure to the harsh environment of the neonatal intensive care unit (NICU), which implies painful procedures as part of life-saving care [5]. The immature nociceptive pathways of the preterm infants undergo significant maturation during the first weeks after birth [6]. Nevertheless, early exposure to pain may interfere with regular brain maturation and developmental neuroplasticity, particularly at the lowest gestational ages (GAs). Repetitive pain exposure can indeed contribute to hyperalgesia and distorted neuroendocrine stress responses, events that are related to altered brain maturation and adverse neurodevelopmental, behavioral, and cognitive consequences, which can persist later in life [6,7,8,9]. Several MRI studies showed that greater pain exposure was associated with reduced regional brain volumes, a more immature white matter microstructure, and altered functional connectivity [10,11,12,13].

Although the American Academy of Pediatrics (AAP) recommends preventing neonatal procedural pain for both ethical reasons and for reducing any potential detrimental consequences [14], at present, there are no universally accepted standardized neonatal pain-management strategies, mainly because of conflicting evidence concerning the efficacy and potential harm of commonly used analgesic agents.

Electroencephalography (EEG) is a non-invasive and sensitive bedside tool that is particularly useful for evaluating the functional status of the newborn’s brain. This can be visually assessed equally from the raw signal or from a time-compressed amplitude-integrated EEG (aEEG) trend display, and both can precociously predict the neurodevelopmental outcomes in preterm neonates [15].

The clinical classification of the spontaneous neonatal EEG background is traditionally based on a visual assessment of multiple features: continuity, quality of sleep wake organization, interhemispheric synchrony (IHS), symmetry, and amplitude [16]. However, due to the lack of objective criteria for classifying the EEG trace and to the absence of any other established method to yield appropriate diagnostic accuracy to support modern neuroscience or medicine, visual EEG interpretation requires a certain amount of expertise.

Computer-assisted analyses of neonatal EEG may serve as physiological biomarkers of neural plasticity and neurodevelopmental outcomes [17,18,19]. Signal processing techniques enable the investigation of neuronal network maturation by characterizing the differentiation and integration of short- and long-range functional connections [17,20]. The corpus callosum is an important long-distance neuronal connector, which links associative areas of both cerebral hemispheres and promotes functional interhemispheric connectivity [21]. During the second half of gestation, cortico-cortical and thalamocortical connections are established and contribute critically to network synchrony and interhemispheric integration [22,23,24].

The Brain Simmetry Index (BSI) is an electrophysiological bivariate measurement of brain activity, applied to quantify the interhemispheric spectral symmetry of the EEG [25]. As this feature is a two-phase synchronization measure (bivariate) [26], it has some limitations in providing a full picture of global interactions in neural systems [27]. To overcome this issue, multivariate measures have been introduced. Circular Omega Complexity (COC) is one of the multivariate measures to evaluate the degree of phase synchronicity. A study performed by Baboukani et al. demonstrated that COC detects inter-hemispheric phase synchrony changes in newborns with a great accuracy [28].

The present study aimed firstly to investigate the impact of pain exposure on EEG features in a group of preterm newborn born < 32 weeks of GA without major brain injury studied at a term equivalent age (TEA); secondly, we assessed if the proposed EEG analysis may be predictive of the neurodevelopmental outcome at 2 years of corrected age (CA) using the Bayley Scales of Infant and Toddler Development, third edition (Bayley-III).

## 2. Materials and Methods

### 2.1. Study Population

In this prospective cohort study, 77 preterm infants born before 32 weeks of GA between September 2018 and May 2021 and admitted to the NICU of Careggi University Hospital in Florence were enrolled. The study was approved by the Pediatric Ethics Committee (approval number 112/2018), and written informed consent was obtained from all parents. Infants with congenital, genetic, or metabolic disorders and those with severe brain injury, defined as intraventricular hemorrhage (IVH) grade ≥ 3 [29] or cystic periventricular leukomalacia (PVL) [30], were excluded.

### 2.2. Perinatal Data

Clinical data were obtained through a daily chart review and the included GA, birth weight, mode of delivery, sex, need for and duration of mechanical ventilation, patent ductus arteriosus (PDA) requiring treatment, bronchopulmonary dysplasia (BPD) [31], postnatal steroid exposure, culture-proven sepsis, necrotizing enterocolitis (NEC) [32], intraventricular hemorrhage (IVH) [29], and periventricular leukomalacia (PVL) [30]. Weight and head circumference z-scores at birth and at the time of the EEG recording were calculated according to the INES growth charts [33].

### 2.3. Pain Evaluation and Management

Exposure to neonatal pain was quantified by recording the number of skin-breaking procedures, including heel lance, endotracheal intubation, peripheral intravenous or central line insertion, intramuscular injection, chest tube insertion, urinary catheterization, and lumbar puncture, from birth to the time of the EEG recording. For subsequent analyses, infants were classified into low (lowest 50%)- and high (highest 50%)-pain-exposure groups based on the median number of painful procedures. This approach allowed stratification of the cohort into groups with relatively lower and higher cumulative burdens of painful procedures, reflecting different levels of clinical stress exposure.

Opioids, including fentanyl and morphine, were administered to mechanically ventilated infants according to pain scores and clinical indications, in line with the guidelines of the Italian Society of Neonatology [34]. Fentanyl was used as a first-line treatment, while morphine was introduced in cases of prolonged ventilator dependence to avoid fentanyl tolerance or tachyphylaxis, typically between the tenth and fourteenth day of fentanyl therapy. Fentanyl was administered either as intravenous boluses (1–3 μg/kg) or continuous infusion (0.5–3 μg/kg/h), as required. Morphine was administered intravenously with a loading dose (0.05–0.1 mg/kg), followed by continuous infusion (0.01–0.05 mg/kg/h), and orally once full enteral feeding was achieved [34]. For statistical analyses, opioid exposure was coded as a dichotomous variable (yes/no).

### 2.4. EEG Recordings

EEG recordings were performed using the NeMus ICU system, Nemus-EB Neuro polygraph and GalNT/EP EXAM software (http://www.ebneuro.com/en/emg/nemus-1, 1 April 2024) (EB Neuro, Florence, Italy), which allows simultaneous acquisition of video-EEG (VEEG), amplitude-integrated EEG, and continuous somatosensory evoked potentials (SEPs) from the same set of electrodes, with signals displayed in on-demand windows [35]. Due to the small head size of preterm infants, 10 pediatric disposable surface electrodes, including reference and ground electrodes, were used for VEEG recordings in accordance with the international 10–20 system. In addition, electrocardiogram, electrooculogram, electromyogram of the mylohyoid muscle, respiratory activity (chest belts), and synchronized video were recorded. For visual EEG analysis, the following 10 bipolar derivations were used: Fp2–C4′, C4′–O2, Fp2–T4, T4–O2, Fp1–C3′, C3′–O1, Fp1–T3, T3–O1, T4–C4′, and C3′–T3. Recordings lasted approximately 1 h to capture a complete spontaneous sleep cycle and awake phase.

### 2.5. Multivariate EEG Functional Connectivity Analysis

Each EEG recording was filtered using a band-pass FIR filter (EBNEURO Nemus Galileo NT 4.5, Florence, Italy) between 1 and 45 Hz. Then, a sub-windowing procedure without overlap was applied to each EEG signal. In other words, each EEG functional connectivity feature was computed for all the sub-windows generated for each subject. During preliminary evaluations, the following window lengths were considered: 4, 8, 16, 32, 64, 128, and 256 s windows. Moreover, after the filtering step, all the windows with a maximum amplitude greater than 500 µV or with at least the 75% of the values equal to 0 µV were excluded a-priori [36]. Thus, to characterize functional connectivity, two EEG-based indices, namely the revised BSI and the COC, were studied.

### 2.6. Revised Brain Simmetry Index (BSI)

In this study, the BSI was considered as proposed by van Putten in van Putten (2007) [25]. Specifically, the version implemented in the NEURAL MATLAB toolbox (Version 0.4.4) was used [37].

The BSI is an estimator of the interhemispheric asymmetry as defined in Equation (1):

(1) $$rBSI\left(t\right)=\frac{1}{K}\sum_{n=1}^{K}|\frac{R_{n}^{*}(t)-L_{n}^{*}(t)}{R_{n}^{*}(t)+L_{n}^{*}(t)}|$$

With $R_{n}^{*}\left(t\right)=\frac{1}{M}\sum_{ch=1}^{M}a_{n}^{2}(ch,t)$,

Where *a_n_* is the Fourier coefficient of the EEG channel *ch* (and *M* the number of channel for each hemisphere considered) evaluated ad time *t*.

The BSI is the absolute value of the relative difference in the average spectral density of the right and left hemispheres in the frequency range from 1 to 25 Hz. Furthermore, the BSI can also be evaluated in a specific frequency range (e.g., for delta brain waves between 1–4 Hz).

In brief, the BSI calculated the mean of the absolute difference at each frequency of all left-sided and corresponding right-sided hemisphere electrode pairs. Next, this left minus right difference at each frequency was divided by their sum and averaged to obtain an index (BSI), ranging from 0 (perfect symmetry) to 1 (maximum asymmetry) [26]. In terms of brain maturation, a decrease in the BSI can be interpreted as a marker of advancing maturational organization, whereas an increase in the BSI reflects a greater hemispheric imbalance, consistent with immature or delayed maturation [38,39], rather than definitive long-term cortical reorganization.

In this work, the epoch on which the BSI was averaged was the entire EEG sub-window, and the same hemispheric channel combination was evaluated (i.e., using M = 1). Moreover, BSI features were also evaluated for the following combination: Fp1-C3 vs. Fp2-C4, considering different EEG frequency ranges.

### 2.7. Circular Omega Complexity (COC)

The multivariate measure considered was the so-called Circular Omega Complexity (COC) originally proposed by Baboukani et al. [28]. The COC assesses the level of phase dependency within multivariate signals by quantifying the dimensionality of the state-space formed by their corresponding instantaneous phases [28] and is applied as an indicator of the strength of local and global connectivity.

Briefly, considering K EEG derivations (in this work, 8 EEG bipolar derivations, excluding T4–C4′, and C3′–T3), the matrix X(K × n) is built: **X** = {x_1_(*n*); …; x_K_(*n*)}, where n are the number of window samples. Then, the so-called circular correlation c^**X**^ is computed between each pair of derivations (*k* and *l*, Equation (2)) considering their instantaneous phase obtained by the Hilbert Transform.

(2) $$c_{k,l}^{X}=\frac{\sum_{n=0}^{N-1}sin(\phi_{k}\left[n\right]-\bar{\phi_{k}})sin(\phi_{l}\left[n\right]-\bar{\phi_{l}})}{\sqrt{\sum_{n=0}^{N-1}{sin}^{2}(\phi_{k}\left[n\right]-\bar{\phi_{k}}){sin}^{2}(\phi_{l}\left[n\right]-\bar{\phi_{l}})}}$$

where $\phi_{k}$ and $\phi_{l}$ are the circular mean of the instantaneous phase. Thus, the circular correlation matrix ${CCM}^{X}$ is defined as ${CCM}^{X}≔[c_{k,l}^{X}{]}_{K\times K}$, where, in our case, *K* = 8. From ${CCM}^{X}$, their eigenvalues $\lambda_{m}$ were extracted, indicating how the derivations are synchronized. The COC varies between 0 and 1, where higher values indicate higher phase synchrony and thus higher connectivity [28]. Because functional integration and phase synchrony among distributed brain regions change with age and the maturation of large-scale neural networks [40,41], variations in COC may be interpreted in a maturational context: a decrease in COC suggests weaker phase coordination and relatively immature network integration, whereas an increase in COC reflects stronger phase synchronization consistent with advancing development of inter-regional cortical networks.

According to Baboukani et al. [28] and Frassineti et al. [42], before the computation of COC, the EEG signals were decomposed applying the Stationary Wavelet Transform (SWT) [43]. The SWT was applied with 5 levels of decomposition to each EEG window and derivation. The mother wavelet function was Symlet 2. For the fs = 128 Hz, this choice allows separating the EEG rhythms in approximation and detail levels. Specifically, only the detail level d1 (approximately representing the frequencies between 32 and 64 Hz) was excluded before the computation of multivariate EEG indexes. Therefore, the coefficients from the detail level d2 (approximately from 16 to 32 Hz) to the approximation level a6 (approximately from 0 to 2 Hz) were considered. In other words, COC indexes were computed for each SWT decomposition level (e.g., COC d2). Moreover, the mean values of all the COC values obtained for all the SWT levels were calculated (hereinafter COC_µ_).

In summary, the following 14 connectivity features were extracted from the EEG recordings: the BSI for each hemispheric pair combination (i.e., Fp2-C4 vs. Fp1-C3, C4-O2 vs. C3-O1, Fp2-T4 vs. Fp1-T3 and T4-O2 vs. T3-O1), the BSI between Fp1-C3 and Fp2-C4 at different brain waves (BSI delta, BSI, theta, BSI alpha, BSI beta), and COC-derived features (mean values (μ) and specific SWT values from d2 to a5 decomposition levels, which correspond to specific spectra bands).

### 2.8. Neurodevelopmental Outcome

The Bayley Scales of Infant and Toddler Development, third edition (BSITD-III), were applied to assess the outcome at 2 years of corrected age (CA). Cognitive and motor composite scores were standardized with a mean of 100 and a standard deviation of 15. The threshold to assess a neurodevelopmental delay was set to below 85 for all scales.

### 2.9. Statistical Analysis

The infants’ clinical characteristics were described as the mean and standard deviation, median and interquartile range (IQR), or rate and percentage. The Student’s *t*-test or Mann–Whitney *U* test was used to compare continuous data, while the *X*^2^ test was used to compare categorical data.

Median values of the connectivity features extracted from the EEG recordings were compared using the Wilcoxon Mann Whitney test.

To evaluate if the functional connectivity features extracted from the EEG recordings at TEA were correlated with the number of painful procedures and later with neurological outcomes at 24 months, multivariate statistical analysis was performed using generalized linear mixed-effects (GLME) models. First, GLME was performed for all the EEG connectivity features previously mentioned. Moreover, during the GLME analysis, the following clinical characteristics were evaluated as effects: GA, post-menstrual age (PMA) at recording, BPD, sex, and fentanyl and morphine exposure.

Lastly, GLMEs were performed to examine the association of Bayley-III scores with the EEG connectivity features, accounting for pain, GA, PMA, gender, and BPD. The categorical features of each Bayley scale were evaluated (e.g., cognitive 24-months: 0 = low scores ≤ 85, 1 = high score).

The normal distribution was used for all the GLMEs, the Maximum pseudo likelihood was used as a method for estimating the model parameters, and the identity function was used as the link function. Before the fit of the GLME models, the outliers for each EEG features were removed from each subject separately, and then, all the remaining observations were normalized (z-scored) across all the subjects. In this work, an element that is greater than a 3 scaled median absolute deviation (MAD) away from the median was considered an outlier.

## 3. Results

Seventy-seven preterm infants with a mean GA of 27 ± 2 weeks were enrolled. The 8-channel EEG recordings were acquired at 39 ± 3 weeks of PMA. The duration of the EEG recordings was 41 ± 14 min. The cumulative median number of painful procedures was 84 (IQR 59–125).

Infants who had more painful procedures had a lower GA and birth weight, had more frequent PDA, BPD, and sepsis, and were more frequently treated with opioids, compared to infants who were less pain-exposed (Table 1). Moreover, they underwent later EEG recordings, exhibiting smaller auxological parameters at that time (Table 1) and, regarding the EEG connectivity measures, displayed lower COC μ, lower COC related to θ, and α bands values, compared to the low-pain group (Table 2).

Bayley-III neurodevelopmental scores at 24 months were collected for 59 infants and, among them, 48 (80%) had a normal cognitive score, and 43 (73%) had a normal motor score. Infants lost to follow-up did not significantly differ from those retained in terms of the GA, major neonatal morbidities, pain exposure, or EEG recording characteristics (all *p* > 0.05).

Statistical results obtained after the GLME analysis are shown in Table 3. Results are related to the 8 sec windows. In detail, only EEG features that presented at least a statistically significant effect (*p* < 0.05) are reported.

### 3.1. Assessment of Pain Exposure and Other Clinical Risk Factors Affecting Connectivity Features Among Preterm Infants

**Figure 1:**
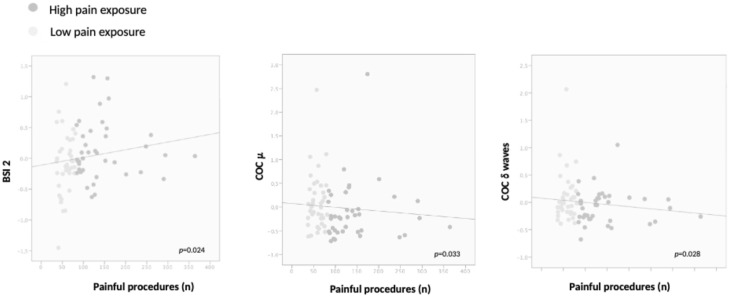
Correlation among BSI 2, COC μ, COC related to δ waves, and pain exposure. Generalized linear mixed-effects (GLME) models R = 0.26, R = 0.35, R = 0.12, respectively.

The GLME revealed that higher pain exposure resulted in higher BSI 2 (C3-O1/C4-O2), lower COC μ, and COC related to δ waves (*p* = 0.024, *p* = 0.033, and *p* = 0.028 respectively; Supplementary Tables S1 and S2, Figure 1). Fentanyl exposure was associated with increased BSI values related to α and β waves (*p* = 0.04 and 0.010, respectively; Supplementary Table S1). The BSI related to α waves was also inversely predicted by PMA (*p* ≤ 0.001) and directly by BPD (*p* = 0.027; Supplementary Table S1). Morphine exposure was associated with greater BSI 2 (C3-O1/C4-O2) values (*p* = 0.032, Supplementary Table S1), and lower COC μ, and COC related to high δ, θ, α, and β wave values (all *p* < 0.05 respectively; Supplementary Tables S2 and S3).

### 3.2. Assessment of Connectivity Features and Neurodevelopment Among Preterm Infants

**Figure 2:**
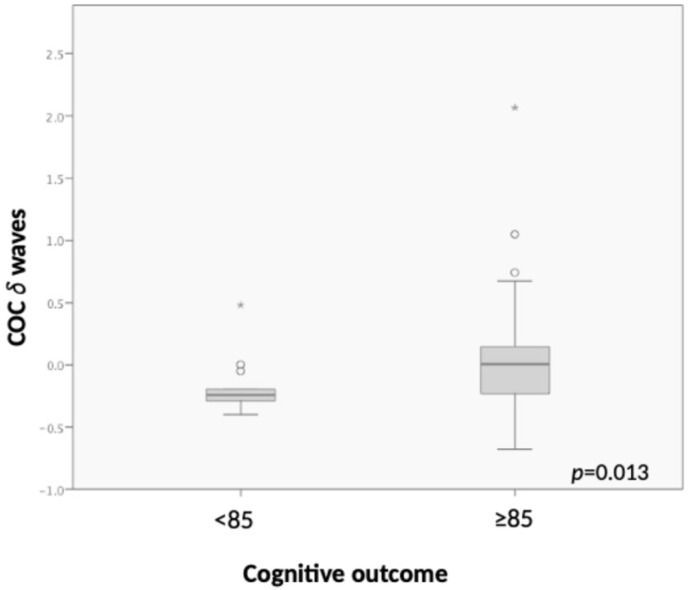
COC low δ wave values in infants with cognitive outcomes > 85 and ≤85. Median and IQR. Generalized linear mixed-effects (GLME) models R = 022. * outliers.

In GLME models, greater COC values related to low δ waves were associated with higher cognitive outcomes (*p* = 0.034; Supplementary Table S4, Figure 2), adjusting for gender, PMA at recording, pain, and BPD. None of the other EEG features explored were associated with Bayley scores.

## 4. Discussion

In this prospective study, in preterm infants without major brain injury, who under-went EEG recordings at TEA, we examined the interaction between early pain exposure, clinical risk factors, including analgesic drug administration, brain connectivity, and the outcome at 2 years CA. We found that greater early-life pain and analgesic drug administration were associated with abnormal EEG connectivity and that abnormal brain connectivity was associated with poorer neurodevelopmental outcomes. These relationships seemed to be independent from other comorbidities, since they remained significant even after adjusting for clinical risk factors.

The second and third trimesters of gestation represent a critical period for the formation and organization of cerebral connectivity, including the development of thalamocortical pathways, the growth of long-range corticocortical fibers, and the subsequent maturation of short-range corticocortical connections [22,44].

At early epoch, the development of synchrony patterns is under the control of the thalamus/brainstem, so it is loose and inconsistent [45]. In fact, synchronous high-amplitude delta wave bursts in the occipital areas can be detected around 26–27 weeks of GA [46]. This pattern is supposed to be under the subcortical–thalamic control because the cortex is still immature and the corpus callosum synaptogenesis is still ongoing [47,48,49]. The development of synchrony patterns from 34 weeks of gestation is thought to be a result of the rapid expansion of callosal connections and of cortical projections [21]. During such a critical phase, preterm infants are exposed to the harsh environment of the NICU where they necessarily undergo painful and stressful procedures, inappropriate noise and light exposure, and other non-physiological inputs [5].

Along with thalamocortical connections, nociceptive systems start to develop between 24 and 28 weeks of gestation, whereas the descending endogenous modulation of the pain pathway only matures close to TEA [50]. Early exposure to painful inputs during the first days of life seem to interfere with brain maturation and with later neurodevelopment in preterm born children. Recent studies reported, indeed, that pain exposure is not only associated with microstructural alterations of the encephalic white matter, including the corpus callosum, but also with smaller thalamic volumes at TEA [13]. Consistent with the volumetric findings, early pain exposure is, in fact, responsible for slower thalamic growth, as well as for microstructural alterations in thalamocortical pathways [13,51].

Recent functional MRI studies have shown that early pain exposure is associated with altered functional connectivity between the thalamus and the somatosensory cortex [12] and with changes in the development of limbic network connectivity, including connections involving the hippocampus and amygdala [52,53].

Specifically, our results showed that higher pain exposure resulted in a higher BSI, and lower COC μ and COC related to δ waves. The BSI is a quantitative measure for assessing the degree of asymmetry between the two hemispheres of the brain and can be considered a measure of discontinuity in a neonatal EEG [26]. COC is a measure of cortical activity and can be applied as a measure of the phase synchrony. It is reported that cortical activity increases with the ongoing maturation, and EEG asymmetry has a downward trend with the increasing of PMA [54]. Our findings showed that both EEG features exhibited a robust correlation with pain exposure and that repeated pain exposure can cause a continuous disruption in cortical activity, leading to altered brain maturation and connections. These results are in agreement with those of Lavanga et al., who displayed the effects of early pain on cortical activity in newborns [55], describing a more dysmature EEG, as evidenced by an increased level of discontinuity in higher-pain-exposed infants.

In preterm infants, pain stimulation induces higher δ activity and δ brushes, distributed over the whole cortex due to underdeveloped cortico-cortical connections [56]. This widespread diffused δ activity is present up to 35 weeks of GA, when a mature cortical response appears [57]. As previously supposed by Lavanga et al., neonatal pain might induce a different brain rhythmicity, especially in terms of the δ band, and might lead to a greater discontinuity of the EEG [55]. Interestingly, our results indicated, specifically, pain as predictor of lower COC values related to δ waves.

Therefore, since pain might induce diffused δ activity [57,58], it could ultimately increase the discontinuous tracing of the cortex, contributing to a more dysmature pattern. Therefore, the higher BSI and the lower COC values are the result of a more dysmature tracing, as a reflection of abnormal cortical maturation.

In our model, fentanyl and morphine administration both influenced the EEG features. Opioids such as morphine and fentanyl are the most used analgesic medications in the NICU and are administered to infants to reduce the pain and stress induced by procedures and mechanical ventilation. Opioid receptors are expressed in the human brain at the cortical level, and the opioid system is crucial for neuronal maturation in light of its modulation of GABAergic synaptic transmission [59].

Nowadays, the impact of opioids on brain maturation and neurodevelopment is not clear [60].

On one hand, randomized controlled trials reported that routine morphine infusion does not influence short-term neurological outcomes [61,62]. On the other hand, morphine exposure has been associated with poorer motor, cognitive, and behavioral outcomes in infants born very preterm [63,64,65,66,67] with the persistence of this association at school age [53,66,67]. Similar findings have been observed with fentanyl exposure in preterm infants, with a greater cumulative dose of fentanyl associated with poorer neurodevelopmental outcomes [53,68,69].

The effect of opioids was also previously described on the EEG in preterm infants. Many authors described a detrimental effect of morphine and fentanyl on early brain activity, inducing a significant EEG depression and an increase in the length of inactivity [70,71].

Our results confirmed these findings, indicating that opioid administration might interfere with cortical activity, inducing a greater BSI and lower COC. Thus, opioids might play a role in determining the development of a more dysmature EEG tracing. However, these results must be taken with caution since, in our population, only 13% of infants received morphine.

Furthermore, our findings showed that BPD was associated with adverse effects on brain connectivity, reflected by increased BSI values. This observation may be explained by two complementary mechanisms. First, BPD is a well-established risk factor for adverse neurodevelopment and has been associated with altered brain maturation even in the absence of overt brain injury [72]. Second, infants who develop BPD typically require prolonged mechanical ventilation [73], which represents a sustained source of stress and repeated painful exposures during a critical period of brain development. In this context, the observed association between BPD and altered connectivity may also reflect the cumulative effects of prolonged ventilation, stress, and pain-related interventions, including opioid administration. Although our statistical models did not demonstrate collinearity with opioid exposure, these factors remain closely intertwined and difficult to fully disentangle in observational studies.

PMA at recording was also a predictor of the BSI. Neonatal EEG patterns change as a function of GA, as well as PMA [74]. This result is supported by previous findings that showed a correlation between early brain activity and PMA. As the PMA increases, the cortical activity tends to rise, whereas EEG asymmetry decreases [54], indicating improved synchronization, enhanced symmetry between hemispheres, and the maturation of brain connectivity [75].

We explored the relationship between EEG features and neurodevelopmental outcomes and found that higher cognitive scores were associated with greater δ-band COC. Previous studies have shown that conventional EEG and amplitude-integrated EEG (aEEG) can predict long-term neurodevelopmental outcomes in preterm infants [15,76,77].

Since higher interhemispheric synchrony is considered a key feature of normal brain development in newborns [16], it is plausible that higher COC values, reflecting greater phase synchrony, are associated with better cognitive outcomes. No other EEG features were significantly associated with Bayley scores. This finding may reflect the characteristics of our cohort, as none of the infants had severe brain injury, only 10% showed low-grade IVH, and most infants exhibited normal Bayley scores at follow-up.

A strength of this study is the application of advanced quantitative EEG connectivity analyses to recordings obtained in a clinically feasible NICU setting. Although EEG acquisition was performed during routine care, the extraction and interpretation of multivariate connectivity metrics require specialized technical expertise and are not yet readily applicable in everyday clinical practice. Nevertheless, this approach provides valuable insights into brain network organization and may inform future efforts aimed at simplifying quantitative EEG tools for translational use.

This study has some limitations. First of all, the small sample size of our population reduces the statistical power of our results, and we cannot, therefore, completely exclude that other factors might influence EEG features. Secondly, an important limitation of this study is the intrinsic difficulty in disentangling the effects of pain exposure from illness severity and prematurity. More immature and clinically unstable infants inevitably undergo a higher number of invasive and painful procedures. Although we adjusted our models for major clinical confounders related to prematurity and illness severity, residual confounding cannot be fully excluded due to the observational nature of the study. However, the association between early-life pain exposure and altered brain development is biologically plausible and supported by converging evidence from experimental animal models [78,79], and the have been reported across multiple independent clinical cohorts using different neuroimaging and neurophysiological techniques, supporting the consistency of these findings [8,9,10,11,12,13,51,52,53,55].

Thirdly, neurodevelopment is also influenced by post-discharge environmental and familial factors, which were not available in this study and may contribute to outcome variability.

An additional limitation is the lack of complementary structural neuroimaging data, such as MRI-based brain morphometry and white and gray matter measures, which could have contributed to a more comprehensive interpretation of the EEG connectivity metrics.

Lastly, in our cohort, only a minority of the infants received morphine and, therefore, we could not draw conclusions on its negative effect.

## 5. Conclusions

Our findings suggest that greater exposure to pain and opioids was associated with reduced brain connectivity in preterm infants. Given the observational nature of the study, these associations should be interpreted with caution. Nevertheless, they highlight the importance of careful and individualized opioid administration based on pain and comfort scores, while considering potential associations with brain connectivity. A further understanding of how opioid exposure relates to brain network organization may provide additional insight into optimal pain-management strategies. COC may represent a useful metric to characterize brain connectivity in relation to neurodevelopmental outcomes. Additional prospective studies based on larger cohorts with longer follow-up are needed to better define the role of pain and opioid exposure and to explore the potential predictive value of connectivity features for brain development.

## Figures and Tables

**Table 1:** Clinical characteristics of studied infants. Mean ± SD, median and (IQR), or rate and (%).

	Low Pain Exposure	High Pain Exposure	*p* Value
Gestational age (wks)	28.9 ± 1.5	26.7 ± 1.7	<0.001
Male	22 (56)	22 (57)	0.143
Birth weight (gr)	1170 ± 271	834 ± 213	<0.001
Birth weight, Z score	0.06 ± 0.87	−0.27 ± 0.85	0.101
Head circumference at birth (cm)	25.5 ± 2.2	23.8 ± 2.1	<0.001
Head circumference, z score at birth	0.06 ± 1.17	−0.41 ± 1.07	0.070
Caesarean section	32 (82)	24 (63)	0.067
Apgar score at 5 min	8 (7–8)	8 (7–8)	0.979
Mechanical ventilation (n)	6 (16)	26 (68)	<0.001
Mechanical ventilation duration (d)	0 (0–0)	3 (0–20)	<0.001
NICU stay (d)	68 ± 21	106 ± 27	<0.001
PMA at EEG recording (wks)	39.0 ± 1.1	39.7 ± 1.0	0.013
Weight at EEG recording (g)	2286 ± 296	2168 ± 347	0.116
Weight z-score at EEG recording	−1.73 ± 1.00	−2.72 ± 1.10	<0.001
Head circumference at EEG recording (cm)	31.9 ± 1.5	31.6 ± 1.7	0.319
Head circumference z-score at EEG r recording	−1.41 ± 1.43	−2.75 ± 1.10	0.023
PDA requiring treatment (n)	16 (41)	34 (89)	<0.001
NEC (n)	0 (0)	1 (2)	0.507
BPD (n)	6 (15)	22 58)	<0.001
Sepsis (n)	13 (33)	26 (68)	<0.001
IVH (n) *grade I* *grade II*	3 (7) 0 (0)	3 (8) 2 (5)	0.408
Postnatal steroids (n)	1 (3)	16 (42)	<0.001
Fentanyl exposure (n) dose (mcg/kg)	20 (51) 6.6 ± 3.1	29 (76) 297.2 ± 45.8	0.017 <0.001
Morphine exposure (n) dose (mg/kg)	1 (3) 0.3 ± 0.1	9 (27) 7.8 ± 1.9	0.006 <0.001
Midazolam exposure (n) dose (mg/kg)	1 (3) 0.03 ± 0.16	16 (42) 0.43 ± 0.50	<0.001 0.022
Cognitive outcome > 85	25 (52)	23 (47)	0.412
Motor outcome > 85	23 (53)	20 (46)	0.360

**Table 2:** Connectivity features of studied infants. Median and (IQR).

	Low Pain Exposure	High Pain Exposure	*p* Value
BSI 2 (C3−O1/C4−O2)	−0.11 (−0.42—0.19)	−0.04 (−0.25–0.36)	0.568
BSI 3 (Fp1−T3/Fp2−T4)	0.03 (−0.15–0.18)	−0.04 (−0.17–0.21)	0.908
BSI 4 (T4−O2/T3−O1)	−0.70 (−0.22–0.09)	−0.02 (−0.15–0.15)	0.304
BSI μ	−0.11 (−0.30–0.18)	−0.03 (−0.22–0.37)	0.304
Connectivity BSI δ band	−0.02 (−0.32–0.16)	−0.03 (−0.16–0.31)	0.908
Connectivity BSI θ band	−0.03 (−0.16–0.06)	−0.04 (−0.11–0.15)	0.568
Connectivity BSI α band	−0.04 (−0.23–0.17)	−0.03 (−0.12–0.21)	0.908
Connectivity BSI β band	−0.07 (−0.32–0.20)	−0.04 (−0.27–0.30)	0.304
COC μ	−0.001 (−0.26–0.39)	−0.21 (−0.51–0.12)	0.030
COC low δ band	−0.02 (−0.18–0.17)	−0.05 (−0.30–0.07)	0.734
COC high δ band	0.002 (−0.23–0.27)	−0.18 (−0.38–0.08)	0.210
COC θ band	0.05 (−0.26–0.30)	−0.29 (−0.44–0.10)	0.033
COC α band	0.03 (−0.27–0.34)	−0.24 (−0.44–0.11)	0.030
COC β band	−0.08 (−0.28–0.41)	−0.20 (−0.41–0.04)	0.210

**Table 3:** GLME analysis between EEG features and clinical risk factors. Statistically significant results.

EEG Features	Clinical Risk Factors with Statistically Significant Effects	Direction of the Effect (Increase/Decrease)	*p* Value
BSI 2 (C3-O2/C4-O2)	pain	↑	0.024
	morphine	↑	0.032
Connectivity BSI α band	PMA at recording	↓	<0.001
	BPD	↑	0.027
	fentanyl	↑	0.043
Connectivity BSI β band	fentanyl	↑	0.010
COC μ	pain	↓	0.033
	morphine	↓	
COC low δ	pain	↓	0.028
COC high δ band	morphine	↓	0.014
COC θ band	morphine	↓	<0.001
COC α band	morphine	↓	<0.001
COC β band	morphine	↓	0.017

## Data Availability

All of the relevant data are within the manuscript.

## Supplementary Materials

The following supporting information can be downloaded at: https://www.mdpi.com/article/10.3390/children13020210/s1. Table S1. GLME model between BSI 2, BSI α band, BSI β band, pain and sedative drugs exposure. Table S2. GLME model between COC μ, COC low δ band, COC high δ band, pain and sedative drugs exposure. Table S3. GLME model between COC θ band, COC α band, COC β band, pain and sedative drugs exposure. Table S4. GLME model between COC low δ band and cognitive outcome at 2 years corrected age (CA).
