# Supplementary material for: Pain, Opioids, and Functional Connectivity in Preterm Infants
Source: Children (Basel). 2026 Jan 31;13(2):210. doi: 10.3390/children13020210 (PMC12940043; doi:10.3390/children13020210)
Supplement: Supplementary file 1 [file children-13-00210-s001.zip › children-4100681-supplementary.pdf]

Supplementary Table 1. GLME model between **BSI 2**, **BSI α band**, **BSI β band**, pain and sedative drugs exposure.

	**BSI 2**<br><br>R2 adjusted 0.26			**BSI α band**<br><br>R2 adjusted 0.35			**BSI β band**<br><br>R2 adjusted 0.22		
	Coefficient	pValue	95% C.I.	Coefficient	pValue	95% C.I.	Coefficient	pValue	95% C.I.
**GA**	0.06	0.127	-0.01-0.13	0.004	0.816	-0.03-0.03	-0.01	0.634	-0.08-0.05
**PMA**	-0.01	0.755	-0.10-0.07	-0.67	**<0.001**	-0.10- -0.03	-0.03	0.402	-0.10-0.04
**Fentanest**	-0.08	0.218	-0.22-0.05	0.06	**0.043**	-0.001-0.12	0.14	**0.010**	-0.03-0.26
**Morphine**	0.20	**0.032**	0.01-0.39	0.02	0.520	-0.05-0.11	0.07	0.341	-0.08-0.24
**Gender**	-0.01	0.806	-0.14-0.10	0.01	0.586	-0.04-0.02	0.015	0.782	-0.09-0.12
**Pain**	0.17	**0.024**	0.02-0.32	0.02	0.422	-0.04-0.09	-0.01	0.821	-0.14-0.11
**BPD**	0.02	0.752	-0.12-0.16	0.07	**0.027**	-0.13-0.007	-0.09	0.150	-0.21-0.03

Supplementary Table 2. GLME model between **COC μ**, **COC low δ band, COC high δ band,** pain and sedative drugs exposure.

	**COC μ** R2 adjusted 0.35			**COC low δ band** R2 adjusted 0.12			**COC high δ band** R2 adjusted 0.28		
	Coefficient	pValue	95% CI	Coefficient	pValue	95% CI	Coefficient	pValue	95% CI
**GA**	-0.02	0.647	-0.10-0.06	-0.02	0.294	-0.08-0.02	-0.01	0.770	-0.08-0.06
**PMA**	-0.06	0.190	-0.15-0.03	-0.04	0.173	-0.10-0.01	-0.04	0.307	-0.12-0.03
**Fentanest**	-0.002	0.968	-0.15-0.14	-0.02	0.588	-0.12-0.06	0.01	0.860	-0.11-0.23
**Morphine**	-0.29	**0.006**	-0.5- -0.08	-0.06	0.344	-0.19-0.06	-0.21	**0.014**	-0.38- -0.04
**Gender**	-0.01	0.805	-0.15-0.11	-0.01	0.818	-0.09-0.07	0.008	0.883	-0.10-0.12
**Pain**	-0.18	**0.033**	-0.34- -0.01	-0.11	**0.028**	-0.22-0.01	-0.13	0.052	-0.2-0.001
**BPD**	-0.04	0.605	-0.19-0.11	-0.01	0.758	-0.11-0.08	-0.001	0.983	-0.13-0.12

Supplementary table 3. GLME model between **COC θ band**, **COC α band, COC β band,** pain and sedative drugs exposure.

	**COC θ band**<br><br>R2 adjusted =0.20			**COC α band**<br><br>R2 adjusted 0.30			**COC β band**<br><br>R2 adjusted 0.37		
	Coefficient	pValue	95% CI	Coefficient	pValue	95% CI	Coefficient	pValue	95% CI
**GA**	-0.001	0.968	-0.07-0.07	0.001	0.958	-0.07-0.07	0.03	0.436	-0.05-0.12
**PMA**	-0.05	0.171	-0.13-0.02	-0.04	0.304	-0.12-0.03	-0.04	0.420	-0.14-0.05
**Fentanest**	0.01	0.661	-0.09-0.15	-0.004	0.942	-0.13-0.12	0.02	0.747	-0.12-0.17
**Morphine**	-0.34	**<0.001**	-0.52- -0.16	-0.35	**<0.001**	-0.53- -0.17	-0.26	**0.017**	-0.48- -0.04
**Gender**	-0.02	0.685	-0.14-0.09	-0.03	0.541	-0.15-0.08	-0.09	0.211	-0.23-0.05
**Pain**	-0.12	0.088	-0.26-0.01	-0.13	0.06	-0.27-0.06	-0.12	0.173	-0.29-0.05
**BPD**	-0.04	0.490	-0.18-0.08	-0.08	0.229	-0.21-0.05	-0.11	0.170	-0.28-0.04

Supplementary Table 4. GLME model between **COC low δ band** and cognitive outcome at 2 years corrected age (CA).

	**Cognitive outcome**<br><br>R2 adjusted 0.22		
	Coefficient	pValue	95% CI
**COC low δ band**	5.03	**0.034**	-27.73-9.85
**GA**	0.51	0.078	0.48-1.10
**PMA**	-0.77	0.779	-0.05-0.32
**Gender**	-0.68	0.173	-1.61-0.31
**Pain**	1.21	0.061	0.02-2.39
**BPD**	0.68	0.141	-0.21-1.63
